# In Vitro Evaluation of Colistin–Meropenem Activity Against XDR and PDR Carbapenemase-Producing *Klebsiella pneumoniae* and *Acinetobacter baumannii*

**DOI:** 10.3390/pathogens15020214

**Published:** 2026-02-13

**Authors:** Shahinda Rezk, Nada Younis Elwakeel, Abeer Ghazal, Amel Elsheredy, Daniel Baecker, Ahmed Noby Amer

**Affiliations:** 1 Microbiology Department, Medical Research Institute, Alexandria University, Alexandria 21561, Egypt; shahinda.rezk@alexu.edu.eg (S.R.);; 2 Institute of Virology, Medical Center, University of Freiburg, 79104 Freiburg, Germany; 3 El Amereya General Hospital, Ministry of Health, Alexandria 23711, Egypt; 4 Department of Pharmaceutical and Medicinal Chemistry, Institute of Pharmacy, Freie Universität Berlin, Königin-Luise-Straße 2+4, 14195 Berlin, Germany; 5 Microbiology and Immunology Department, Faculty of Pharmacy and Drug Manufacturing, Pharos University in Alexandria, Canal El Mahmoudia Street, Beside Green Plaza Complex, Alexandria 21648, Egypt

**Keywords:** carbapenem-resistance, *Acinetobacter baumannii*, *Klebsiella pneumoniae*, colistin, meropenem, combination therapy

## Abstract

Carbapenem-resistant *Klebsiella pneumoniae* (CRKP) and *Acinetobacter baumannii* (CRAB) pose significant therapeutic challenges due to their high resistance and global spread. Combination therapy with colistin (COL) and meropenem (MEM) was used to enhance antimicrobial activity. This study evaluated the COL-MEM combination against CRKP and CRAB isolates with a high resistance profile. A total of 58 carbapenem-resistant clinical isolates (31 CRKP and 27 CRAB), including extensively resistant and pandrug-resistant strains, were collected over a period of 12 months. Synergy between COL and MEM was assessed by microdilution checkerboard (MCB) and time-kill (TKA) assays. Carbapenemase genes were detected using molecular methods. The results showed that the COL-MEM combination yielded synergy (35.5% and 40.7%, respectively) and additive effects (35.5% and 37.0%, respectively), while no antagonism was observed. TKA confirmed bactericidal activity, especially at doubled MCB-detected concentrations, indicating dose-dependent activity. The significant reduction in the minimum inhibitory concentration in the combination indicated its potential for dose optimization, minimizing COL-associated toxicities. Genotypic profiling showed that the expression of *bla_NDM_* and *bla_Oxa-48_* can reduce synergy. These findings, obtained with isolates of high resistance, support the efficacy of this combination therapy and could reduce the dose-related side effects of COL. However, they also highlight genotype-specific variations and COL resistance mechanisms as limiting variables.

## 1. Introduction

A global threat to human health is posed by antimicrobial-resistant ESKAPE pathogens (*Enterococcus faecium*, *Staphylococcus aureus*, *Klebsiella pneumoniae*, *Acinetobacter baumannii*, *Pseudomonas aeruginosa*, and *Enterobacter* species). Gram-negative ESKAPE group pathogens are classified as “Priority 1: Critical” by WHO [1,2]. These bacteria greatly affect the disease burden in developing and developed countries and are commonly associated with multiple life-threatening infections [3]. As a result, ESKAPE bacterial infections are a leading cause of mortality and morbidity worldwide. The acquisition of antimicrobial resistance (AMR) genes by ESKAPE pathogens has limited the treatment choices for serious infections, expanded the burden of disease, and elevated treatment-failure-linked death rates [4,5,6].

Due to their limited treatment choices and high attributable morbidity and death, carbapenem-resistant *Klebsiella pneumoniae* and *Acinetobacter baumannii* (CRKP and CRAB) are considered among the highest-priority Gram-negative bacteria and are important causes of healthcare-associated infections globally. Alarmingly high rates of carbapenem resistance and widespread carriage of carbapenemase genes have been documented by recent surveillance and molecular studies [7,8].

Combination therapy is usually used to widen the empiric coverage produced by two antibiotics with different activities, to utilize the synergy observed in vitro between two antibiotic agents compared to one (to improve clinical outcome), or to avoid or postpone the rise of resistance during treatment [9]. In addition, monotherapy frequently fails with XDR/PDR organisms. Combination therapy provides complementary or synergistic killing, therefore allowing lower doses of each agent to be used. In the case of colistin (COL), such dose-sparing strategies may help to mitigate nephrotoxicity while maintaining or even improving the bactericidal effect [10].

It is reported that COL has been used in combination with other antimicrobial agents such as tigecycline, gentamicin, meropenem (MEM), or fosfomycin to treat drug-resistant bacterial infections [11]. Many healthcare providers support the use of the COL–carbapenem combination for the treatment of carbapenem-resistant Gram-negative bacterial infections. This is due to the strong in vitro synergy between both antimicrobials [12].

Given the generally beneficial approach of combining COL with the carbapenem antibiotic MEM and due to their synergy, as documented in the above-mentioned studies, the aim of the present work was to determine the effect of COL–carbapenem combination for the treatment of infections associated with CRKP and CRAB isolates with high resistance profiles.

## 2. Materials and Methods

This study included a total of 58 carbapenem-resistant isolates, 31 CRKP (K1–K31) and 27 CRAB isolates (A1–A27). The isolates were collected from the Medical Research Institute Hospital Laboratory, Alexandria University, over a one-year period from April 2022 to March 2023.

### 2.1. Isolate Identification and Susceptibility Testing

All isolates were identified and tested for antimicrobial susceptibility using the VITEK^®^ 2 COMPACT system (bioMérieux, Nürtingen, Germany), a laboratory machine used for identifying and testing the susceptibility of bacteria. The isolates were determined regarding their susceptibility pattern. In accordance with Magiorakos et al. [13], multidrug-resistant (MDR) isolates were defined as having acquired non-susceptibility to at least one agent in three or more antimicrobial categories. If non-susceptibility to at least one agent in all but two or fewer antimicrobial categories existed, the isolate was termed as extensively drug-resistant (XDR). Pandrug-resistant (PDR) isolates were specified as having non-susceptibility to all agents in all antimicrobial categories.

The minimum inhibitory concentration (MIC) of MEM and COL for the 58 isolates included in the current study was confirmed by the broth microdilution method. Broth dilution was carried out in 96-well microtiter plates. Bacteria were inoculated into a Müller–Hinton broth in the presence of different concentrations of an antimicrobial agent. Growth was assessed after incubation for a defined period of time (18 h), and the MIC was determined as the lowest concentration of the assayed antimicrobial agent that inhibits visible growth. MEM and COL results were interpreted based on the performance standards of the Clinical and Laboratory Standards Institute (CLSI) [14].

### 2.2. Microdilution Checkerboard Assay

The microdilution checkerboard (MCB) assay is a method to determine the effects of antibiotic combinations. Different concentrations of two antibiotics are used along the rows and columns of the microtiter plate in order to determine the MIC for each antibiotic in combination. MIC determination is performed by visible turbidity readings [15,16]. All MCB experiments were performed in duplicate in Müller–Hinton broth. The final concentration range in the checkerboard plate was obtained for COL (8 → 0.125 µg/mL vertically) and MEM (256 → 0.5 µg/mL). The cumulative fractional inhibitory concentration (FIC) index (ΣFIC) represents a dimensionless index and was calculated according to Equation (1),

ΣFIC = FIC(MEM) + FIC(COL)(1)

where the FIC of each antibiotic is equal to the concentration of the antibiotic in the effective combination divided by its MIC as a single agent. If the ΣFIC index is ≤0.5, the combination is considered synergistic. If the ΣFIC is >0.5 to ≤1, then it is considered an additive effect, ΣFIC >1 to ≤4 is interpreted as indifference, and ΣFIC >4.0 is interpreted as antagonism [17,18]. All ΣFIC values were reported to two decimal places.

### 2.3. Time-Kill Assay

Five CRKP and five CRAB isolates that showed synergy for the combination were used to perform time-kill assays (TKA). Only five isolates per species were selected due to the labor-intensive nature of the assay. However, they represented the genotypic patterns of the isolates. Several concentrations were tested against the selected isolates, including the MIC for COL and MEM alone, the concentrations of both antibiotics in combination that showed the lowest ΣFIC in MCB (1× MIC), and double these concentrations (2× MIC). All TKA experiments were performed in duplicate in 50 mL sterile plastic Falcon conical tubes. A standard inoculum (10^6^ colony-forming units (CFU) per mL) was added to a total volume of 20 mL Müller–Hinton broth containing the appropriate concentrations of the tested antibiotics at the beginning of the experiment. The Falcon tubes were loosely capped before incubation. A shaking incubator was used at 200 rpm in the experiment to ensure the optimization and uniformity of aeration and growth. Sub-culturing was performed from each broth at different time intervals (2, 4, 6, 12, and 24 h), followed by detection of the bacterial count [17]. After colony counting, the results were charted on regular graph paper with the colony count converted to the decadic logarithm (log_10_) on the *y*-axis and time on the *x*-axis in an arithmetic scale [19]. The bactericidal effect was calculated as

(number of log_10_ CFU/mL of drug combination at a time point) − (number of log_10_ CFU/mL of starting inoculum at time point) (2)

Negative values indicated a lower CFU from the starting inoculum. Bactericidal activity is defined as a ≥3 log_10_ CFU/mL reduction in viable bacterial count from the initial inoculum at a specified time point.

The synergetic effect was calculated as

(number of log_10_ CFU/mL of drug combination at a time point) − (number of log_10_ CFU/mL of the most active single agent at time point) (3)

Negative values indicated a lower CFU number with the checkerboard MIC. Synergy in TKA is defined as a ≥2 log_10_ CFU/mL greater reduction in bacterial count by the antibiotic combination compared with the most active single agent at the same time point.

### 2.4. Genotypic Detection of Carbapenemase Genes

Molecular detection of selected carbapenemase genes (New-Delhi-metallo-β-lactamase gene (*bla_NDM_*), OXA-48-Type Oxacillinase (*bla_Oxa-48_*), *K.-pneumoniae*-carbapenemase gene (*bla_KPC_*), Verona-integron-encoded metallo-β-lactamase gene (*bla_VIM_*), and Imipenemase gene (*bla_IMP_*) was carried out using a conventional polymerase chain reaction (PCR). Bacterial deoxyribonucleic acid (DNA) was extracted using the boiling method. Conventional PCR was performed for all isolates using the Cosmo “Hot Start” PCR RED Master Mix (Willowfort, Birmingham, UK). The thermal profile was as follows: initial denaturation 95 °C/10 min (one cycle), 40 cycles of denaturation at 95 °C for 30 s, annealing for 30 s at a temperature specific to the primers used, extension at 72 °C, extension time specific to the amplicon size of the target genes (see Appendix A, Table A1). Agarose gel electrophoresis was performed on Mupid-EXU^®^ (ADVANCE Co., Ltd., Tokyo, Japan) for the identification of a specific band of the genes. The DNA bands were visualized on a UV transilluminator (Herolab, Wiesloch, Germany).

### 2.5. Statistical Analysis

Data were analyzed using the R platform in RStudio (version 4.4.2). Descriptive and analytical statistics were performed. Categorical data were summarized as numbers and percent (%), while quantitative data were summarized as the median, interquartile range (IQR), minimum, and maximum. The Wilcoxon rank sum test was used to test non-parametric data between two independent groups. Chi-square test was used to test categorical data. Univariate logistic regression with a calculated odds ratio was applied to test the effect of carbapenem-resistance genes on the synergetic effect.

## 3. Results

### 3.1. Resistance Pattern

This study included 31 CRKP and 27 CRAB isolates. These isolates showed high levels of resistance to the tested antibiotics. All of them were resistant to carbapenem, with MEM MIC range of 8–256 µg/mL with *K. pneumoniae* and 8–128 µg/mL with *A. baumannii*.

COL resistance was detected in 7 among the 27 *A. baumannii* strains (25.9%), which is the lowest resistance rate among all tested antibiotics, with the MIC ranging from 0.125 to 8 µg/mL. For *K. pneumoniae*, COL resistance was detected in 25 of 31 isolates (80.6%), with a MIC range from 0.125 to 512 µg/mL. The resistance pattern for the studied *K. pneumoniae* isolates included 19 XDR (61.3%) and 12 PDR (38.7%) isolates, while for *A. baumannii*, 25 isolates were XDR (92.6%) and 2 were PDR (7.4%).

### 3.2. Microdilution Checkerboard Assay

**Figure 1:**
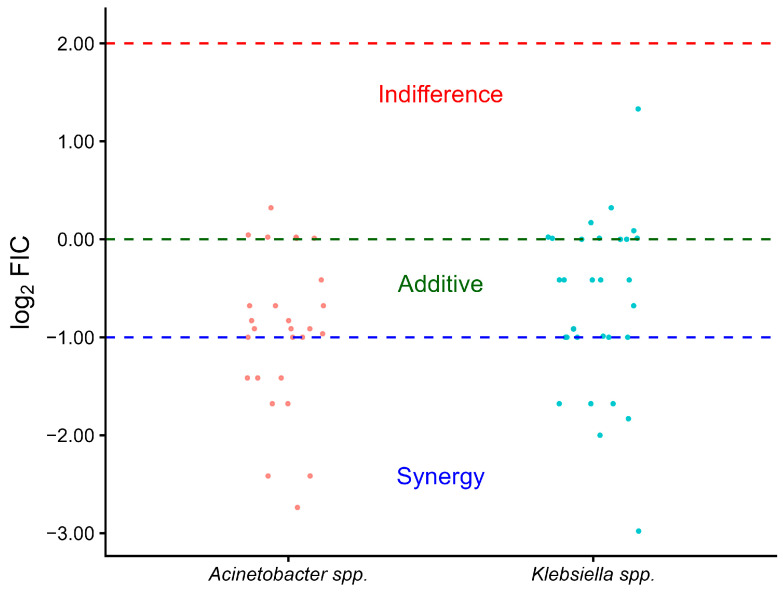
Representation of the log_2_-transformed ΣFIC values on *y*-axis for the illustration of indifference, additive effects, or distinct synergy patterns between CRAB (red dots) and CRKP (cyan dots).

The MCB assay results of the combination with CRKP isolates, as calculated from the ΣFICs, showed synergism in 11 isolates (35.5%), an additive effect in another 11 isolates (35.5%), and indifference in 9 isolates (29.0%). The tested combination with the investigated CRAB isolates exhibited synergism in 11 cases (40.7%) and an additive effect in 10 isolates (37.0%). Among the 27 isolates, 6 (22.2%) exhibited indifference. No antagonism was detected (Table 1). The combination showed that the majority of the CRAB isolates were distributed around the synergy threshold, while CRKP isolates display greater variability, with a larger proportion clustering near the additive and in the indifference zone (Figure 1).

**Figure 2:**
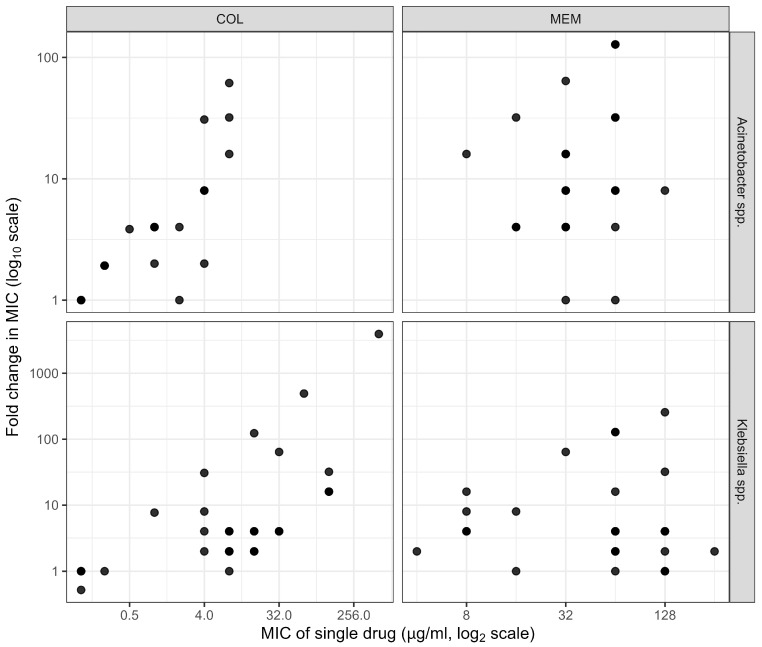
Representation of the log–log scatter plot of fold changes in MIC values (*y*-axis, log_10_ scale) versus the MIC of each single drug (*x*-axis, log_2_ scale) for colistin (COL) and meropenem (MEM). It illustrates the magnitude of MIC reduction for combining both drugs.

Although true synergy (ΣFIC ≤ 0.50) was observed in 35.5% and 40.7% (Appendix A, Table A2) of CRKP and CRAB isolates, respectively, the combination showed a highly significant (*p*-value < 0.001) reduction in the MICs of MEM (range = 0.5–128 µg/mL) and COL (range = 0.125–8 µg/mL) when applied in combination compared to the MIC of each single drug (MEM: 4–256 µg/mL, COL: 0.125–512 µg/mL) (Table 2). It was observed that among CRKP isolates, higher MICs tend to correspond to greater fold reductions, especially with COL, indicating a potentially strong synergistic rescue effect in highly resistant strains. This pattern is less prominent among CRAB isolates (Figure 2).

### 3.3. Time-Kill Assay

The results showed that none of the checkerboard MICs of the combination achieved the bactericidal effect with all of the selected CRKP isolates, while the double checkerboard MICs exhibited the bactericidal effect in all tested isolates. In all CRAB isolates, the combination showed bactericidal activity in the checkerboard MIC except for isolate A18. Applying the double checkerboard MICs, bactericidal effects were obtained by all investigated CRAB isolates (Appendix A, Table A3).

Synergy was calculated considering the differences in bacterial count from the most active single agent at each point of the time-kill curve. The results showed that the combination at 1× MIC achieved transient synergy only with two isolates (i.e., K4 and K13) at 2 h, while at 2× MIC all selected isolates showed synergy until the final time point, except for K7, which could not be evaluated during the period from 2 h to the final time point.

**Figure 3:**
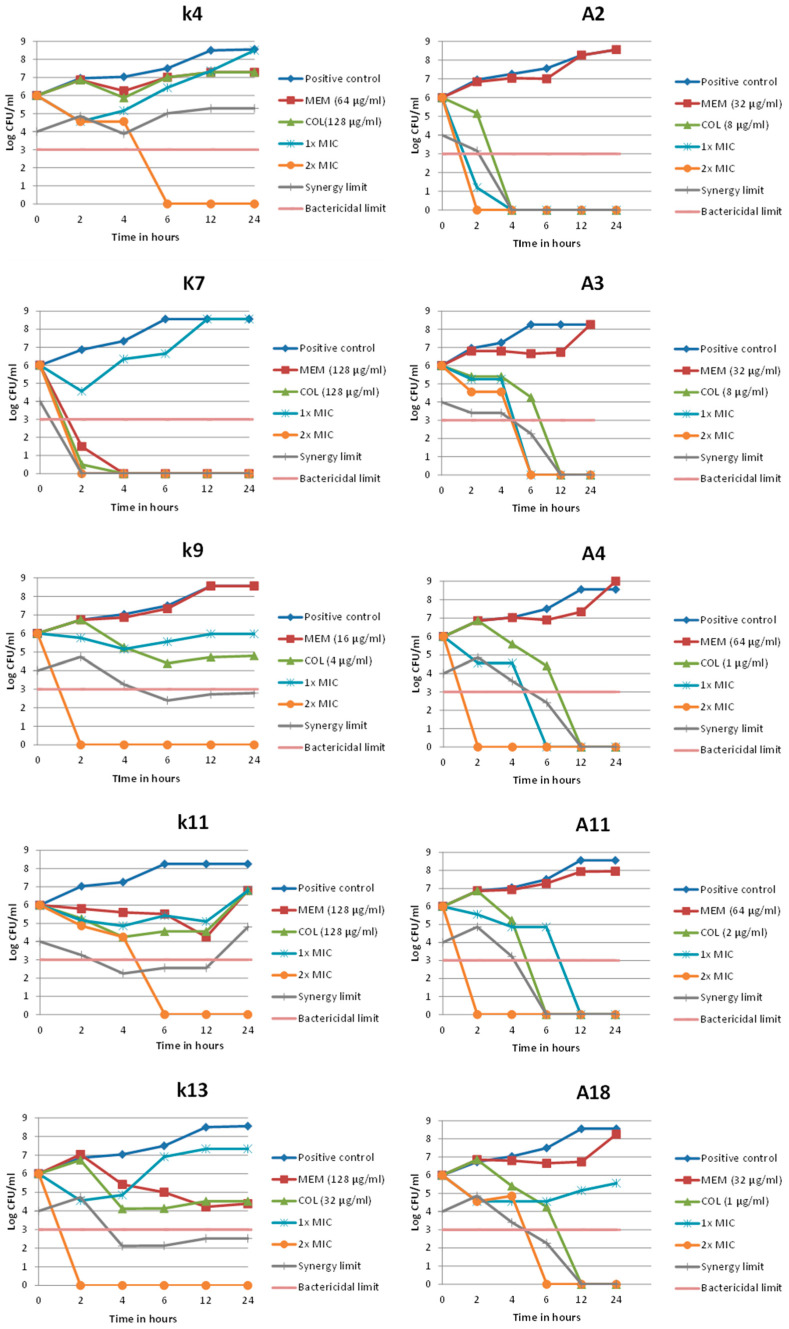
Time-kill curves using the single drugs MEM and COL as well as their checkerboard combination (1× MIC, 2× MIC) against selected isolates of *K. pneumoniae* (K4, K7, K9, K11, K13) and *A. baumannii* (A2, A3, A4, A11, A18). Each time-kill curve covered five time points (2, 4, 6, 12, 24 h).

Different patterns of synergy were observed among the CRAB isolates at both concentrations of the combination (1× MIC and 2× MIC). For all isolates, synergy was obtained at different time points. However, for both concentrations, the reduction in CFU was below the limit of detection (LOD), thus suggesting a reduction close to sterilization. Therefore, no synergy could be assessed at the final time point, which does not generally negate the synergistic effect of the combination (Figure 3).

The study findings demonstrate a dose-dependent effect of the combination, with marked differences in synergistic activity observed between colistin-intermediate (COL-I) and colistin-resistant (COL-R) CRKP isolates. A significant decrease in the calculated ΣFIC was observed for CRKP isolates resistant to COL (*p*-value = 0.047). Moreover, there was a significant correlation between COL-resistant isolates and the synergistic (*p* < 0.001) or additive (*p* = 0.03) effects of the combination, respectively. Concerning the CRAB isolates, there was no significant difference between the two groups, except for significant additive effects with COL-intermediate isolates (*p* = 0.01) (Table 3).

### 3.4. Genotypic Detection of Carbapenem-Resistance Genes

Three different carbapenemase genes were identified among the investigated isolates. The prevalence of each gene was detected among the study isolates. The gene *bla_NDM_* was the most prevalent carbapenemase gene (100% in *K. pneumoniae* and 92.6% in *A. baumannii*), followed by *bla_Oxa-48_* (87.1% in *K. pneumoniae*, and 100% in *A. baumannii*), while *bla_VIM_* was found in only 12.9% of *K. pneumoniae* isolates. However, *bla_KPC_* and *bla_IMP_* were not detected in any of the isolates. Among our tested isolates, *bla_NDM_* and *bla_Oxa-48_* were co-detected in 74.2% of CRKP and 92.6% of CRAB isolates. In addition, 12.9% of CRKP isolates were found to harbor three carbapenemase-resistance genes (*bla_NDM_*, *bla_Oxa-48_*, and *bla_VIM_*) (Appendix A, Table A4).

The effect of the presence of the carbapenemase genes *bla_Oxa-48_*, *bla_VIM_*, and *bla_NDM_* on the potential to exhibit synergy is summarized in Table 4. The odds ratios for the occurrence of no synergy among the CRKP isolates bearing the *bla_Oxa-48_* gene and among the CRAB isolates with the *bla_NDM_* gene were 2.00 and 1.50, respectively. As these values exceed the threshold of 1, the probability of interplay between the genes and the occurrence of no synergy is elevated. The odds ratio among CRKP isolates in the presence of the *bla_VIM_* gene was 0.14, thus limiting the probability of a correlation between the gene and the biological behavior regarding synergy.

## 4. Discussion

The threat of AMR is rising at a tremendous rate, and the situation may be aggravated in developing countries due to gross abuse of antimicrobials [20]. The current study evaluated the COL-MEM combination against CRKP and CRAB strains with high resistance profiles.

The resistance profile of these isolates adduced in the present work is different regarding the percentage of multidrug resistance compared to similar studies [21,22,23,24,25], given different conditions (use of antibiotics and occurrence of infections, among other factors).

The rising AMR in Egypt was clearly obvious in this study. The results showed extremely high MICs especially among CRKP isolates against both COL and MEM (0.125–512 µg/mL and 8–256 µg/mL, respectively), which is much higher than the MICs reported in several studies such as those by Brennan-Krohn et al. and Yu et al. which revealed much lower MICs for both COL (2–16 µg/mL and 2–32 µg/mL, respectively) and MEM (1–32 µg/mL and 4–256 µg/mL, respectively) [26]. In CRAB isolates, the MIC range for MEM was 8–128 µg/mL and for COL it was 0.125–8 µg/mL.

The main resistance mechanism against treatment with carbapenems in Gram-negative bacteria is the production of β-lactamases (carbapenemases) [27,28]. The most effective carbapenemases, in terms of carbapenem hydrolysis and geographical spread, are *Klebsiella-pneumoniae*-carbapenemase (KPC), Verona-integron-encoded metallo-β-lactamase (VIM), imipenemase (IMP), New-Delhi-metallo-β-lactamase (NDM), and oxacillinase (OXA-48). The production of carbapenemase has a clinical impact since these enzymes hydrolyze almost all β-lactam antibiotics, thus causing high levels of carbapenem MICs [29].

In the current study, we aimed to detect five carbapenem-resistance genes, i.e., *bla_NDM_*, *bla_Oxa-48_*, *bla_KPC_*, *bla_VIM_*, and *bla_IMP_*. The results revealed that *bla_NDM_* was the highest detected carbapenem-resistance gene among *K. pneumoniae isolates* (31/31, 100%), followed by *bla_Oxa-48_* (27/31, 87%), and *bla_VIM_* (4/31, 12.9%). In contrast, the genes *bla_KPC_* and *bla_IMP_* were not found in any isolate of the current study.

Several studies have investigated the prevalence of carbapenem-resistance genes, and different results were reported. According to the study by Pourgholi et al. that aimed to analyze the carbapenemase genes among 71 CRKP isolates, they reported that the most frequent gene was *bla_Oxa-48_*, which was found in 48/71 (67.6%) isolates, followed by *bla_VIM_*, which was detected in 28/71 (39.4%) isolates. The genes *bla*_IMP_, *bla*_NDM_, and *bla*_KPC_ were identified in 19/71 (26.8%), 13/71 (18.3%), and 5/71 (7.0%) isolates, respectively [30]. In a study conducted by Mantazana et al. on 87 CRKP isolates, the phenotypic detection of β-lactamases showed that 73.5% were metallo-β-lactamases (MBL) producers, and 14.9% were KPC producers. However, they failed to mention the type of MBL detected [31].

Regarding CRAB isolates in our study, the gene *bla_Oxa-48_* was detected in all (27/27, 100%) isolates, followed by the gene *bla_NDM_* (25/27, 92.6%). However, the genes *bla_KPC_*, *bla_VIM_*, and *bla_IMP_* were not detected in any of the investigated isolates. In line with the current research work, in a study in Iran on *A. baumannii* isolated from a burn wound infection, the gene *bla_Oxa-48_* was detected in 46/50 (92.0%) cases [32].

Interestingly, CDC (Centers for Disease Control and Prevention) testing in the Antibiotic Resistance Laboratory Network during 2019 found that carbapenemase genes were detected in 83% of CRAB isolates. Most CRAB isolates possess genes for carbapenemases that have been specifically identified among *Acinetobacter* species. These more common genes make OXA-23-like, OXA-24/40-like, and OXA-58-like oxacillinases. However, the following genes, i.e., *bla_KPC_*, *bla_IMP_*, *bla_NDM_*, *bla_VIM_*, and *bla_Oxa-48-like_*, were rarely isolated from CRAB [33,34]. This reflects the unusual genetic combination of the current study isolates.

The development of new antibiotics is far too slow. The scarcity of effective therapeutic agents has encouraged the trial to combine existing agents for synergistic activities against drug-resistant bacteria [21].

In our study, synergy testing for the COL-MEM combination was carried out using two different assays, MCB followed by TKA, which are considered the standard methods for the investigation of antimicrobial combination [31].

Regarding the CRKP isolates, the MCB results for the COL-MEM combination were as follows: 35.5% synergy with a ΣFIC range = (0.1269–0.5 µg/mL), 35.5% additive (ΣFIC range = 0.5–1 µg/mL), and 29.0% indifference (ΣFIC range = 1.007–2.516 µg/mL). Much lower MIC ranges were obtained in combination for MEM (0.5–128 μg/mL) and COL (0.125–8 μg/mL) compared to each single agent, i.e., MEM (4–256 µg/mL) and COL (0.125–512 µg/mL).

Studies of COL-MEM synergy report highly variable results among CRKP, ranging from very low to very high rates of synergy, reflecting differences in isolate selection and testing methods. For example, Brennan-Krohn et al. found only 15% synergy (3/20) among XDR and COL- and carbapenem-resistant isolates [16]. Also, a study conducted by Atalla et al. observed mixed results for five tested isolates with the COL-MEM combination (three synergy, one additive, and one indifferent) [35]. On the other hand, Ontong et al. reported 81.8% synergy (9/11) in MDR *K. pneumoniae*, Goel et al. found 88.0% (44/50) in MDR CRKP, and Yu et al. reported 65.0% synergy among MEM- and COL-resistant CRKP [21,26,36].

On the other hand, CRAB isolates undergoing the MCB assay of the combination resulted in 40.7% synergy (ΣFIC range = 0.15–0.5 µg/mL), 37.0% additive effects (ΣFIC range = 0.512–0.75 µg/mL), and 22.2% indifference (ΣFIC range = 1.007–1.25 µg/mL) with much lower MIC ranges for MEM (0.5–64 μg/mL) and COL (0.125–2 μg/mL), compared to those of the single-agent MEM (8–128 µg/mL) and COL (0.125–8 µg/mL).

Multiple in vitro studies report highly variable COL-MEM synergy rates with CRAB isolates, consistent with the heterogeneous results observed in our study. For example, Ju et al. found 30.0% synergy in MDR CRAB using MCB at much lower combination concentrations than single-agent MICs [23]. Daoud et al. reported 54.5% synergy (6/11) among MEM-resistant, COL-intermediate *A. baumannii* [37]. Pongpech et al. and Le Minh et al. observed higher synergy rates of 73.3% and 68.0%, respectively [38,39]. Mantzana et al. reported very high synergy (73.6% for XDR *K. pneumoniae* and 99.0% for XDR *A. baumannii*) but used a fixed-ratio MIC strip method rather than standard checkerboard or time-kill assays [31].

These discrepancies likely reflect differences in local resistance backgrounds and resistance phenotypes (MDR vs. XDR/PDR), tested concentration ranges, and combination methods and underscore the need for standardized assays and genotype-aware interpretation when comparing synergy data across studies.

Mechanistically, COL damages the outer membrane, which increases the effectiveness of MEM against CRKP and CRAB. Also, if a mixed population contains cells resistant to MEM or to COL, the partner drug can kill that subpopulation. For instance, COL can eradicate minor MEM-resistant mutants while MEM kills any COL-resistant survivors This complementary killing also yields additive/synergistic effects [31,40]. This was obvious among CRAB isolates and COL-intermediate isolates, where the combination showed better effects for the combination with a significant difference in the additive effect. Regarding the CRKP isolates, the combination achieved a better performance among COL-resistant isolates. A lower ΣFIC was noted and significantly higher rates of synergy and additive effects were observed among the COL-resistant isolates. Other studies reported similar observations of a high rate of synergy with COL resistance [21,35]. However, in the previously mentioned study by Yu et al. [26] it was reported that the combination of COL-MEM had better synergistic and additive effects on COL-intermediate isolates than COL-resistant isolates. This statement is opposed to the fact that most of their test isolates were COL-resistant (37/40, 92.5%), and yet they showed a relatively high synergy rate for the MEM-COL combination (26/40, 65.0%). Interestingly, in the study conducted by Mantzana et al. [31] on COL-resistant isolates of *A. baumannii* and *K. pneumoniae*, they exhibited higher synergy rates among *K. pneumoniae* isolates (99.0%) than *A. baumannii* (73.6%).

The discrepancy between *K. pneumoniae* and *A. baumannii* regarding the effect of COL sensitivity on the outcome of the combination may be attributed to the resistance mechanism of COL. In *Klebsiella* and other *Enterobacteriaceae*, the common COL-resistance pathways such as *mcr* or *mgrB/pmr/pho* modifications reduce the bactericidal effect of COL but do not completely abolish its effect on the outer membrane, allowing COL to continue acting as a permeabilizer that enhances MEM penetration and therefore promotes synergy in COL-resistant isolates. In contrast, *A. baumannii* frequently acquires high-level COL resistance through *lpxA/lpxC/lpxD*-associated loss of lipooligosaccharides or specific *pmrCAB* mutations, which eliminate the binding of COL to the target and therefore abolish its permeabilizing and bactericidal activity, making synergy far less likely in COL-resistant strains and more commonly seen only when isolates remain COL-intermediate [41,42].

In the current study, TKA of COL–MEM combinations was performed on five representative isolates per species in the MCB assay. These isolates displayed synergy for the combination and represented the genotypic pattern of the isolates. For the CRAB isolates, the combination achieved 80.0% bactericidal activity at the MIC determined from the MCB assay, while with all five isolates, bactericidal activity was reached at 2× MIC. Moreover, each CRAB isolate demonstrated synergistic effects at one or more time points when tested at both 1× and 2× MIC. By comparison, Ju et al. reported that, in their TKA of 10 CRAB isolates, the COL–MEM combination at 1× MIC produced 100% bactericidal activity, with synergy observed in 50.0% of isolates at 12 h and 40.0% at 24 h [23].

While for CRKP isolates TKA results showed that none of the MIC concentrations exhibited bactericidal effects. However, in 2 out of the 5 (40.0%) isolates transient synergy was achieved at 2 h, but bacterial growth was detected again over time. At 2× MIC concentrations, the bactericidal effect was reached at 6 h after incubation for all of the selected isolates, while in 4 out of the 5 (80.0%) isolates the combination had synergistic effects 6 h after incubation.

In the previously mentioned study by Goel et al. on 39 CRKP isolates, synergy for the studied combination was shown. Among their tested isolates, growth was completely inhibited after 4 h of incubation. The results were the same after 6 h, except for a few colonies counted in the approach using 1/8× MIC. Synergy was maintained after 12 and 24 h. In addition, re-growth was observed but only with low concentrations of the combination. Less than 50 colonies were found at 1/4× and 1/8× MIC of both MEM and COL after 12 h [36]. The pattern shown in the TKA suggested that the combination is concentration-dependent, where a higher dose improved bactericidal and synergistic effects and reduced re-growth risks. This pattern aligns with the mechanism of COL, rapid, dose-driven membrane damage that potentiates MEM, contrasting the time-dependent β-lactam action of MEM alone [43,44]. This highlights the primary player in this combination, which is considered to be COL as the most active single agent.

The kinetics of the TKA showed for both CRKP and CRAB that the colony count may increase after an initial decrease. In line with that, according to the previously discussed study by Lagerback et al., it was also reported that adequate initial killing followed by re-growth was observed with COL alone in all experiments. They added that re-growth is well known to occur in susceptible Gram-negative bacteria exposed to COL, presumably caused by the selection of pre-existing resistant subpopulations, de novo mutations, adaptive resistance, or formation of persister cells [45].

Moreover, Yu et al. reported that re-growth of some strains in the presence of antibiotic combinations (COL-MEM and COL-AK; amikacin) might be due to the selection of the resistant subpopulations through antimicrobial pressure in TKA [26].

The difference in the synergy figures of the present study and other related studies reveals how isolates pertaining to the same species have the ability to show different responses to antibiotics. It should be taken into consideration that the resistance profile and the genetic profile of the test isolates might affect the results of synergy testing.

Most of the previously mentioned studies reported higher synergy rates than in the current research work, which may be attributed to the relatively high percentage of PDR isolates in the current study, compared to published related studies, which stated that most of their isolates were either MDR [21,46], or XDR [23], and none of them reported the presence of PDR isolates [16].

The window for two antibiotics to potentiate one another narrows with increasing resistance, particularly PDR. As a result, this affects the ability of the combinations to reach the synergy threshold, which significantly lowers the total synergy rate [31].

Comparing the study results to some of the published literature, it was obvious that the prevalence of *bla_Oxa-48_* and *bla_NDM_* in CRAB and CRKP isolates was much higher in the current study (*bla_NDM_* 92.6 and 100%, and *bla_Oxa-48_* 100% and 87.0%, respectively). The high prevalence of *bla_Oxa-48_* and *bla_NDM_* among our CRKP and CRAB isolates may reflect regional epidemiology and selective sampling of XDR/PDR isolates. The outcomes showed high statistical significance for no synergy among CRKP isolates carrying the *bla_Oxa-48_* gene and CRAB isolates harboring the *bla_NDM_* (odds ratio 2 and 1.5, respectively). This indicates that the presence of the *bla_Oxa-48_* or *bla_NDM_* genes reduces the probability of having a synergistic effect for the COL-MEM combination.

This could be explained by MEM deactivation by high-level carbapenemases like NDM or OXA-48 [47,48], undermining the synergy associated with the COL permeabilizing effect, especially if the MEM MICs are very high. However, *bla_VIM_* did not show a similar effect. This may reflect the small sample size for VIM-positive isolates in this study (12.9% among CRKP isolates only). The current interpretation is considered exploratory because of the limited number of isolates and limited carbapenemase genes tested in addition to the lack of clear statistical significance. Consequently, these results rather generate hypotheses.

In line with that, Lagerback et al. elicited that the recommendation about implementing the COL-MEM combination for the treatment of carbapenemase-positive *K. pneumoniae* is based on results from studies that comprise mostly KPC- or VIM-producing strains and may not be valid for NDM producers. Additionally, they stated that MEM had little additive effect on all of their isolates, which were all NDM producers [45]. It was also reported that the COL-MEM combination is sometimes recommended for the treatment of infections by mostly KPC- or VIM-producing strains and may not be of much effect for NDM-1 producers [45,49]. In agreement with that, the study conducted by Yu et al. on CRKP isolates showed higher synergy rates (65.0%) for the COL-MEM combination. However, their isolates displayed a different genotypic pattern from ours. Most of their isolates harbored *bla_KPC_* (92.5%), while only 13.5% of isolates expressed *bla_NDM_* [26]. In addition, in a study by Mantazana et al., they found that the highest synergy rate was observed with KPC-producing strains. Synergy rates of 66.7%, 90.9%, and 80.0% were observed for MBL, KPC, and MBL + KPC strains, respectively [31].

Since COL-induced nephrotoxicity has been considered to be dose-dependent [50], it was also suggested that COL-induced neurotoxicity is related to the COL dose and the infusion rate [51]. Therefore, using a smaller concentration of COL in combination with a carbapenem might reduce the dose-related side effects of COL, while achieving a positive outcome.

In the current study, both CRKP and CRAB showed a highly significant reduction in the MICs of MEM (range = 0.5–128 μg/mL) and COL (range = 0.125–8 μg/mL) in combination compared to the MIC of MEM alone (range = 4–256 μg/mL) and COL alone (range = 0.125–512 μg/mL) (*p*-value < 0.001). Moreover, our results documented additive rates of 35.5% and 37.0% among CRKP and CRAB isolates, respectively. The current study showed that combination therapy may offer greater benefit in isolates with elevated MICs, particularly among CRKP, and reinforce the species-specific nature of synergistic interactions.

According to the study conducted by Soudeiha et al., combining COL with carbapenems resulted in a decrease in COL MICs (2-fold reduction) [46]. This study demonstrated that the combination had a positive (additive and synergistic) effect, even on COL-resistant isolates, on 71.0% and 77.7% of CRKP and CRAB, respectively.

Therefore, these study results may be very promising regarding using lower COL concentrations in combination to maximize bacterial killing and minimize the rise of resistance against COL and adverse effects. The current study also showed that the combination still had a synergistic outcome with CRKP even in COL-resistant isolates, which may reflect that COL sensitivity may not be required in *K. pneumoniae*, as COL can act to increase permeability.

These findings are endorsed by several in vivo studies that confirm the superiority of COL-MEM combination over COL monotherapy. A study in Cairo, Egypt, conducted by Abdelsalam et al. revealed the superiority of COL-MEM combination therapy over COL monotherapy in the treatment of MDR *K. pneumoniae*-induced hospital-acquired pneumonia or ventilator-associated pneumonia [24].

Moreover, according to a study by Katip et al., it was reported that in critically ill patients with CRAB infections, combined treatment with COL plus MEM was associated with lower mortality when compared with COL monotherapy. Furthermore, combination therapy was associated with higher clinical and microbiological responses compared to monotherapy with COL [52].

Our study findings are based on in vitro assays and therefore the observed synergy may not directly translate into clinical efficacy, as host factors, pharmacokinetics, and infection site dynamics were not assessed. Moreover, the overall number of isolates was adequate to explore general synergy patterns, while the sample size for certain carbapenemase genotypes remains relatively small, limiting statistical power for genotype–synergy association analyses. In addition, the study isolates were collected from a single geographic region (Alexandria, Egypt), which may limit the ability to generalize the results to other epidemiological settings. Larger, multi-center studies incorporating in vivo or clinical outcome data are warranted to validate these findings. The characterization of colonies emerging at different time points was beyond the scope of this study and should be addressed in future investigations.

The findings of the study support the case for targeted combination therapy based on carbapenemase genotypes and local resistance epidemiology. Such an approach could be effective through application of robust antimicrobial stewardship programs that integrate molecular diagnostics, local surveillance data, and evidence-based prescribing practices. Similar in vitro evidence highlighted that the rational, evidence-based use of COL-containing combinations may optimize antimicrobial utilization and help to preserve last-resort agents like COL in regional hotspots for antimicrobial resistance [53,54,55,56,57].

## 5. Conclusions

In conclusion, in vitro analysis of 31 CRKP and 27 CRAB isolates with a high resistance profile showed that COL-MEM combination therapy yields an intermediate rate of synergy (35.5% and 40.7%, respectively) and additive effects (35.5% and 37.0%, respectively), while no antagonism was observed. TKA confirmed bactericidal activity, particularly at doubled MCB-detected concentrations. Significant MIC reductions (*p* < 0.001) suggested the potential for dose optimization to minimize COL-associated toxicities. Genotypic profiling indicated that the expression of *bla_NDM_* and *bla_Oxa-48_* may attenuate synergy. These findings, with high XDR and PDR rates, support combination therapy as a strategy against the studied pathogens; however, they also highlight genotype-specific variations. Future studies are required to investigate the effect of COL-resistance mechanisms on the combination outcome. In addition, in vivo and clinical studies are essential to validate these results and guide tailored interventions in AMR hotspots.

## Figures and Tables

**Table 1:** Summary of the results obtained with all isolates in the microdilution checkerboard assay under consideration of the IQR of the cumulative fractional inhibitory concentration (ΣFIC), reported in µg/mL.

	CRKP (*n* = 31)			CRAB (*n* = 27)		
Pattern *	Number (%)	ΣFIC Median (IQR)	Range	Number (%)	ΣFIC Median (IQR)	Range
Synergy	11 (35.5%)	0.31 (0.29–0.50)	0.126–0.500	11 (40.7%)	0.38 (0.25–0.50)	0.150–0.500
Additive	11 (35.5%)	0.75 (0.58–0.75)	0.504–1.00	10 (37.0%)	0.56 (0.53–0.63)	0.512–0.750
Indifference	9 (29.0%)	1.01 (1.01–1.13)	1.00–2.52	6 (22.2%)	1.02 (1.01–1.03)	1.01–1.25

* ΣFIC index ≤0.5 is considered synergy, ΣFIC >0.5 to ≤1 is considered as an additive effect, and ΣFIC >1 to ≤4 is interpreted as indifference.

**Table 2:** Comparison between the MICs of COL and MEM when applied alone as single drugs or in combination with each other. The asterisk * indicates a statistically significant difference between the groups (<0.05, Wilcoxon–Mann–Whitney test).

MIC (µg/mL)		Single Drug	Combination COL + MEM	*p*-Value
*K. pneumoniae*, *n* = 31				
COL	Median (IQR)	8.0 (4.0–32.0)	2.0 (0.2–6.0)	<0.001 *
	Range	0.125–512	0.125–8.00	
MEM	Median (IQR)	64.0 (24.0–128.0)	16.0 (2.0–32.0)	<0.001 *
	Range	4.00–256	0.5–128	
*A. baumannii*, *n* = 27				
COL	Median (IQR)	1.0 (0.2–3.0)	0.1 (0.1–0.4)	<0.001 *
	Range	0.125–8.00	0.125–2.00	
MEM	Median (IQR)	32.0 (32.0–64.0)	4.0 (2.0–8.0)	<0.001 *
	Range	8.00–128	0.5–64	

**Table 3:** Comparison of checkerboard assay results regarding the fractional inhibitory concentration (ΣFIC) and the IQR between colistin-intermediate (COL-I) and colistin-resistant (COL-R) CRKP and CRAB isolates. The asterisk * indicates statistical significance, ^a^: Wilcoxon test, ^b^: Chi-square test with Monte Carlo correction.

			COL-I	COL-R	*p*-Value
CRKP *n* = 31	ΣFIC	Median (IQR)	1.0 (0.7–1.0)	0.5 (0.5–1.0)	0.047 ^a^*
		Indifference, *n* = 9	4 (44.4)	5 (55.6)	1 ^b^
		Additive, *n* = 11	2 (18.2)	9 (81.8)	0.03 ^b^*
		Synergy, *n* = 11	0 (0.0)	11 (100.0)	<0.001 ^b^*
CRAB *n* = 27	ΣFIC	Median (IQR)	0.5 (0.5–0.7)	0.4 (0.2–0.8)	0.39 ^a^
		Indifference, *n* = 6	4 (66.7)	2 (33.3)	0.696 ^b^
		Additive, *n* = 10	9 (90.0)	1 (10.0)	0.01 ^b^*
		Synergy, *n* = 11	7 (63.6)	4 (36.4)	0.365 ^b^

**Table 4:** The impact of carbapenemase genes in CRAB and CRKP isolates on the synergistic effect of the MEM-COL combination. The data are given as the absolute number (percentage) of isolates showing synergy/no synergy (i.e., indifference and additive effects). The odds ratios (considering a 95% confidence interval (CI)) represent the correlation between occurrence of the genes and synergy/no synergy.

		Synergy	No Synergy	Odds Ratio (95% CI)
CRKP *n* = 31	*bla_Oxa-48_*			
	negative, *n* = 4	2 (50.0%)	2 (50.0%)	-
	positive, *n* = 27	9 (33.3%)	18 (66.7%)	2.00 (0.21–19.04, *p* = 0.521)
	*bla_VIM_*			
	negative, *n* = 27	8 (29.6%)	19 (70.4%)	-
	positive, *n* = 4	3 (75.0%)	1 (25.0%)	0.14 (0.01–1.28, *p* = 0.110)
CRAB *n* = 27	*bla_NDM_*			
	negative, *n* = 2	1 (50.0%)	1 (50.0%)	-
	positive, *n* = 25	10 (40.0%)	15 (60.0%)	1.50 (0.05–40.98, *p* = 0.783)

## Data Availability

The dataset is available upon request from the corresponding author A.N.A.

## Abbreviations

AMR	Antimicrobial resistance
*bla_NDM_*	New-Delhi-metallo-β-lactamase
*bla_Oxa-48_*	OXA-48-Type oxacillinase
*bla_KPC_*	*Klebsiella-pneumoniae*-carbapenemase
bla_VIM_	Verona-integron-encoded metallo-β-lactamase
bla_IMP_	Imipenemase
bp	Base pair
CFU	Colony-forming units
CI	Confidence Interval
CLSI	Clinical and Laboratory Standards Institute
COL	Colistin
COL-I	Colistin-intermediate
COL-R	Colistin-resistant
CRAB	Carbapenem-resistant *Acinetobacter baumannii*
CRKP	Carbapenem-resistant *Klebsiella pneumoniae*
FIC	Fractional inhibitory concentration
IQR	Interquartile range
LOD	Limit of detection
MBL	Metallo-β-lactamase
MCB	Microdilution checkerboard assay
MEM	Meropenem
MIC	Minimum inhibitory concentration
MDR	Multidrug-resistant
PCR	Polymerase chain reaction
PDR	Pandrug-resistant
TKA	Time-kill assay
XDR	Extensively drug-resistant

## Appendix A

**Table A1:** Primers used to test the carbapenem-resistance genes, with expected amplicon size in base pairs (bp).

Carbapenemase Gene	Primer Sequence (5′→3′)		Annealing Temperature/Time	Amplicon Size/Extension Time	Reference
*bla_NDM_*	Forward	GGTTTGGCGATCTGGTTTTC	52 °C/30 s	621 bp/40 s	[58]
	Reverse	CGGAATGGCTCATCACGATC			
*bla_Oxa-48_*	Forward	GCGTGGTTAAGGATGAACAC	50 °C/30 s	438 bp/30 s	[59]
	Reverse	CATCAAGTTCAACCCAACCG			
*bla_KPC_*	Forward	TGTCACTGTATCGCCGTC	50 °C/30 s	331 bp/25 s	[60]
	Reverse	TATTTTTCCGAGATGGGTGAC			
*bla_VIM_*	Forward	TCTACATGACCGCGTCTGTC	49 °C/30 s	748 bp/45 s	[61]
	Reverse	TGTGTCTTTGACAACGTTCGC			
*bla_IMP_*	Forward	CTACCGCAGCAGAGTCTTTG	50 °C/30 s	587 bp/35 s	[62]
	Reverse	AACCAGTTTTGCCTTACCAT			

**Table A2:** Results of the microdilution checkerboard assay for the COL-MEM combination against CRKP and CRAB isolates.

Isolate	MIC of Single Agent (µg/mL)		MIC of Combination (µg/mL)		Fold Reduction		ΣFIC	Interpretation
	MEM	COL	MEM	COL	MEM	COL		
K1	128	8	32	4	4	2	0.75	Additive
K2	8	64	1	0.125	8	512	0.1269	Synergy
K3	64	16	4	4	16	4	0.3125	Synergy
K4	64	128	16	8	4	16	0.3125	Synergy
K5	64	16	64	8	1	2	1.125	Indifference
K6	64	32	16	8	4	4	0.5	Synergy
K7	128	128	32	4	4	32	0.281	Synergy
K8	64	8	32	2	2	4	0.75	Additive
K9	16	4	2	0.5	8	8	0.25	Synergy
K10	64	0.25	0.5	0.25	128	1	1.0078	Indifference
K11	128	128	32	8	4	16	0.3125	Synergy
K12	64	8	32	4	2	2	1	Additive
K13	128	32	32	8	4	4	0.5	Synergy
K14	64	8	16	4	4	2	0.75	Additive
K15	64	8	16	8	4	1	1.25	Indifference
K16	32	0.125	0.5	0.25	64	0.5	2.516	Indifference
K17	128	8	4	4	32	2	0.53	Additive
K18	8	4	2	2	4	2	0.75	Additive
K19	128	4	64	0.125	2	32	0.531	Additive
K20	256	16	128	8	2	2	1	Additive
K21	16	16	16	0.125	1	128	1.0078	Indifference
K22	128	8	32	2	4	4	0.5	Synergy
K23	8	16	2	4	4	4	0.5	Synergy
K24	8	4	2	1	4	4	0.5	Synergy
K25	128	512	128	0.125	1	4096	1.0004	Indifference
K26	64	0.125	0.5	0.125	128	1	1.007	Indifference
K27	128	0.125	0.5	0.125	256	1	0.5039	Additive
K28	8	0.125	0.5	0.125	16	1	1.0625	Indifference
K29	128	32	128	0.5	1	64	1.0156	Indifference
K30	64	32	32	8	2	4	0.75	Additive
K31	4	1	2	0.125	4	1	0.625	Additive
A1	16	4	4	0.5	4	8	0.375	Synergy
A2	32	8	4	0.5	8	16	0.1875	Synergy
A3	32	8	4	0.25	8	32	0.15	Synergy
A4	64	1	8	0.25	8	4	0.375	Synergy
A5	32	1	8	0.25	4	4	0.5	Synergy
A6	32	1	8	0.25	4	4	0.5	Synergy
A7	64	1	16	0.25	4	4	0.5	Synergy
A8	64	2	0.5	2	128	1	1.0078	Indifference
A9	32	4	32	0.125	1	32	1.03125	Indifference
A10	64	4	8	2	8	2	0.625	Additive
A11	64	2	8	0.5	8	4	0.375	Synergy
A12	32	1	2	0.5	16	2	0.5625	Additive
A13	8	4	0.5	0.5	16	8	0.1875	Synergy
A14	32	0.5	2	0.125	16	4	0.3125	Synergy
A15	32	0.25	4	0.125	8	2	0.625	Additive
A16	32	0.125	8	0.125	4	1	1.25	Indifference
A17	32	0.125	0.5	0.125	64	1	1.0156	Indifference
A18	32	1	2	0.25	16	4	0.3125	Synergy
A19	64	0.25	2	0.125	32	2	0.5312	Additive
A20	16	0.25	0.5	0.125	32	2	0.5312	Additive
A21	64	0.25	2	0.125	32	2	0.5312	Additive
A22	32	0.25	2	0.125	16	2	0.5625	Additive
A23	16	0.25	4	0.125	4	2	0.75	Additive
A24	64	0.125	0.5	0.125	128	1	1.007	Indifference
A25	32	0.25	4	0.125	8	2	0.625	Additive
A26	128	0.25	16	0.125	8	2	0.5125	Additive
A27	64	8	64	0.125	1	64	1.0156	Indifference

**Table A3:** The bactericidal and synergistic effect of MEM-COL combination on selected CRKP (*n* = 5) and CRAB isolates (*n* = 5) isolates.

	Isolate	Synergism	Antimicrobial Effect	Sampling Time									
				2 h		4 h		6 h		12 h		24 h	
				1× MIC	2× MIC	1× MIC	2× MIC	1× MIC	2× MIC	1× MIC	2× MIC	1× MIC	2× MIC
*K. pneumoniae* (CRKP)	K4	+	B	−1.44	−1.44	−0.84	−1.44	−0.43	−6.00 **	1.37	−6.00 **	2.50	−6.00 **
			S	−2.30 *	−2.30 *	−0.72	−1.32	−0.58	−7.01 *	0.08	−7.29 *	1.21	−7.29 *
	K7	−	B	−1.44	−6.00 **	0.34	−6.00 **	0.64	−6.00 **	2.25	−6 **	2.56	−6.00 **
			S	4.56	<LOD	6.34	<LOD	6.64	<LOD	8.25	<LOD	8.56	<LOD
	K9	+	B	−0.24	−6.00 **	−0.84	−6.00 **	−0.44	−6.00 **	−0.03	−6.00 **	−0.027	−6.00 **
			S	−0.98	−6.73 *	−0.097	−5.26 *	1.17	−4.39 *	1.25	−4.72 *	1.18	−4.79 *
	K11	+	B	−0.84	−1.14	−1.14	−1.75	−0.57	−6.00 **	−0.9	−6.00 **	0.80	−6.00 **
			S	−0.10	−0.40	0.61	<LOD	0.87	−4.56 *	0.54	−4.56 *	<LOD	−6.80 *
	K13	+	B	−1.44	−6.00 **	−1.14	−6 **	0.91	−6.00 **	1.34	−6.00 **	1.34	−6.00 **
			S	−2.18 *	−6.73 *	0.74	−4.12 *	2.77	−4.14 *	2.81	−4.527 *	2.81	−4.53 *
*A. baumannii* (CRAB)	A2	+	B	−4.8 *	−6.00 *	−6.00 *	−6.00 *	−6.00 *	−6.00 *	−6.00 *	−6.00 *	−6.00 *	−6.00 *
			S	−3.96 *	−5.16 *	<LOD	<LOD	<LOD	<LOD	<LOD	<LOD	<LOD	<LOD
	A3	+	B	−0.74	−1.44	−0.74	−1.44	−6.00 *	−6.00 *	−6.00 *	−6.00 *	−6.00 *	−6.00 *
			S	−0.15	−0.85	−0.15	−0.85	−4.26 *	−4.26 *	<LOD	<LOD	<LOD	<LOD
	A4	+	B	−6.00 **	−1.44	−6.00 **	−1.44	−6 **	−6.00 **	−6.00 **	−6.00 **	−6.00 **	−6.00 **
			S	−6.86 *	−2.30 *	−5.58 *	−1.03	−4.41 *	−4.41 *	<LOD	<LOD	<LOD	<LOD
	A11	+	B	−6.00 **	−0.44	−6.00 **	−1.14	−6.00 **	−1.14	−6.00 **	−6.00 **	−6.00 **	−6.00 **
			S	−6.86 *	−1.30	−5.22 *	−0.36	<LOD	4.86 *	<LOD	<LOD	<LOD	<LOD
	A18	+	B	−1.44	−1.44	−1.44	−1.14	−1.44	−6.00 **	−0.84	−6.00 **	−0.44	−6.00 **
			S	−2.30 *	−2.30 *	−0.85	−0.54	0.30	−4.26 *	5.16	<LOD	<LOD	<LOD

Table showing the difference between the initial count and the count at each time point. B = bactericidal effect; S = synergistic effect. * The results show a synergistic effect. ** The results show a bactericidal effect. <LOD = the count in both the combination and the most active single agent was not detected at this time point.

**Table A4:** The prevalence of carbapenemase genes among the *K. pneumoniae* (*n* = 31) and *A. baumannii* (*n* = 27) isolates.

Gene	*K. pneumoniae*		*A. baumannii*	
	Number	Percentage	Number	Percentage
*bla_NDM_*	31	100%	25	92.6%
*bla_oxa-48_*	27	87.1%	27	100%
*bla_KPC_*	0	0%	0	0.0%
*bla_VIM_*	4	12.9%	0	0.0%
*bla_IMP_*	0	0%	0	0.0%
